# Importance of E/e’ and BNP for early detection of late cardiotoxicity in long-term follow-up of childhood hematologic cancer survivors: a retrospective cross-sectional study

**DOI:** 10.1186/s40959-025-00354-1

**Published:** 2025-06-19

**Authors:** Yuri Okazoe-Hirakawa, Kimikazu Yakushijin, Keiji Kurata, Sakuya Matsumoto, Hiroya Ichikawa, Rina Sakai, Taku Nose, Shiro Kimbara, Yoshiaki Nagatani, Taiji Koyama, Yumiko Inui, Yohei Funakoshi, Naomi Kiyota, Mitsuhiro Ito, Keiko Hatazawa, Hidekazu Tanaka, Nobuyuki Yamamoto, Hironobu Minami

**Affiliations:** 1 Division of Medical Oncology/Hematology, Department of Medicine, Kobe University Hospital and Graduate School of Medicine, 7-5-2 Kusunoki-cho, Chuo-ku, Kobe, Hyogo Japan; 2 Division of Cardiovascular Medicine, Department of Medicine, Kobe University Hospital and Graduate School of Medicine, 7-5-2 Kusunoki-cho, Chuo-ku, Kobe, Hyogo Japan; 3 Department of Pediatrics, Kobe University Graduate School of Medicine, 7-5-2 Kusunoki-cho, Chuo-ku, Kobe, Hyogo Japan

**Keywords:** Childhood cancer survivors, Hematopoietic stem cell transplantation, Late complications, Cardiotoxicity

## Abstract

**Background:**

Childhood cancer survivors (CCS) often develop late complications after their primary disease is cured. Cardiovascular disease is one of the most frequent and serious complications that significantly affects prognosis and quality of life. Early detection and appropriate intervention are expected to improve their prognosis. However, the risk factors for late cardiotoxicity in CCS are not well defined, and biomarkers that can detect cardiac dysfunction prior to the development of heart failure have not yet been established.

**Methods:**

Medical records of childhood hematologic cancer survivors referred to our department for transitional care between January 2016 and October 2023 were reviewed for this cross-sectional study. The relationships between the most recent cardiac function at the review and history of cancer treatment were analyzed.

**Results:**

This study included 34 patients and the median elapsed time since cancer diagnosis was 16.5 years (range, 5–30 years). None of the patients had symptomatic cardiac complications. The E/e’ ratio was significantly higher in survivors with a history of hematopoietic stem cell transplantation (HSCT) than in those who did not undergo HSCT (median, 8.4% vs. 6.25%, *P* = 0.040), while no intergroup differences were observed in ejection fraction (EF), global longitudinal strain (GLS), or the brain natriuretic protein (BNP) level. In addition, the E/e’ ratio was positively correlated with years elapsed since cancer diagnosis (ρ = 0.38, P = 0.034). While there was no clear correlation between years since cancer diagnosis and the BNP level in the overall cohort, a strong correlation was found in patients with a history of HSCT (ρ = 0.73; *P* < 0.01). No significant differences were observed in EF, E/e’ ratio, GLS, and BNP level by cumulative anthracycline dose or history of chest irradiation.

**Conclusions:**

In this study, no patient had late symptomatic cardiac complications. However, in those who had survived for a long time since their cancer diagnosis, particularly those with a history of HSCT, there were significant elevations in the E/e’ ratio and the BNP level. Continuous follow-up is required to determine whether these abnormalities lead to symptomatic cardiotoxicity and whether they serve as useful markers for the early detection of cardiac complications.

## Introduction

Advances in treatment have improved survival rates for childhood cancer patients, and almost 80% of children and adolescents diagnosed with cancer become long-term survivors [1]. However, childhood cancer survivors (CCS) often develop late complications as a result of the numerous therapeutic agents used to treat their cancer [2]. Late toxicity caused by chemotherapy and radiation therapy (RT) may affect multiple organ systems, can be potentially life-threatening, and affect their quality of life [2]. Among these, cardiovascular disease (CVD) has been reported to be the leading cause of mortality in CCS after cancer recurrence and secondary malignancies [3]. Anthracyclines use and RT are significant risk factors for CVD. It has been reported that approximately 10% of pediatric patients who have received anthracyclines at a dose of 300 mg/m^2^ or more develop symptomatic cardiotoxicity [3]. For RT, the cumulative incidence of radiation-induced cardiac disease is estimated to be 10–30% by 5 to 10 years after treatment [4]. Due to these backgrounds, CCS are 15 times more likely to develop heart failure and eight times more likely to die from CVD than the general population [3]. In addition to anthracyclines and RT, allogeneic hematopoietic stem cell transplantation (HSCT) has been reported to increase late cardiac toxicity in adult cancer survivors [5]. This may be due to the high-dose chemotherapy and total-body irradiation used as the conditioning regimen, as well as immunosuppressants or corticosteroids as prophylaxis or treatment for graft-versus-host disease (GVHD) [6]. In pediatric oncology, HSCT is a crucial curative treatment for high-risk hematologic malignancies and solid tumors [7]. Although HSCT sometimes leads to death from complications due to its potent toxicity, there are a growing number of long-term survivors who have had HSCT in childhood because of improvements in treatment outcomes. However, the effect of HSCT on late cardiac toxicity in CCS is unclear.

As stated above, most CCS experience one or more late effects, including cardiac toxicity, due to cancer treatment; thus, most CCS require life-long follow-up care. In response, transitional care from pediatric- to adult-focused departments has become widespread in recent years [8]. For the management of CCS who become adults, appropriate assessment of the risk of late toxicity based on treatment history is required and an adequate follow-up system should be established. In particular, since late cardiotoxicity has a significant impact on prognosis and quality of life, early detection and appropriate intervention are required to prevent the worsening of cardiac complications. In adults, global longitudinal strain (GLS) has been reported to be a useful parameter for the early detection of cancer therapy-related cardiac dysfunction (CTRCD) [9, 10]. However, the long-term utility of GLS in CCS is unclear, and useful biomarkers for the early detection of late cardiotoxicity in this setting have not been identified.

The main purpose of the present study was to evaluate the long-term cardiac function in adult survivors of childhood hematologic cancer and the late cardiac effects of cancer treatment to adequately assess the risk of late cardiotoxicity, and to identify useful parameters for early detection of cardiac dysfunction to reduce the number of CCS whose quality of life is impaired or who die due to late cardiotoxicity.

## Methods

### Patient cohort

In this retrospective single-center study, CCS who had received any cancer treatment for hematological malignancies that were diagnosed before the age of 18 years, and were referred to the Department of Medical Oncology/Hematology, Kobe University Hospital for transitional care between January 2016 and October 2023 were included. Patients who had a relapse of the primary malignancy were excluded.

### Clinical data collection

Information on patient background, primary cancer diagnosis, and treatment history were obtained by reviewing the medical records. The cumulative dose of anthracycline was converted to a doxorubicin equivalent according to the following relative cardiotoxicity (doxorubicin: 1, epirubicin: 0.8, daunorubicin: 0.6, idarubicin: 5, and mitoxantrone: 10.5) [11]. Data from the following laboratory tests and echocardiography that were performed most recent to the time of review were obtained: ejection fraction (EF), E/e’ ratio, GLS, and brain natriuretic peptide (BNP) level. GLS was assessed using 2D speckle-tracking strain analyses (AutoSTRAIN; TOMTEC ARENA, TOMTEC Imaging Systems GmbH, Munich, Germany). Optimal apical two-, three- and four-chamber views were selected, and the software automatically traced the left ventricular endocardium and calculated the GLS. If automatic tracing was deemed to be inaccurate, it was corrected manually. When it was not possible to obtain an appropriate three-pattern view, one strain or the mean of two available strains was adopted.

### Statistical analysis

Characteristics of the study cohort and data from laboratory tests and echocardiography were summarized as median (range). These parameters were compared by patient characteristics or treatment history using the Mann–Whitney U test or the Kruskal–Wallis test. In comparison by cumulative dose of doxorubicin, the patients were assigned to one of three groups: <100 mg/m^2^, 100–249 mg/m^2^, and ≥ 250 mg/m^2^, based on the risk classification of the European Society of Cardiology (ESC) guidelines [11]. Correlations between cardiac parameters and age or years elapsed since cancer diagnosis were determined using the Spearman’s rank correlation coefficient. For all analyses, statistical significance was set at *P* < 0.05. All analyses were performed using EZR version 1.68 [12].

## Results

### Cohort

The study cohort included 34 patients (18 females [53%]). The demographic and clinical characteristics of the patients are summarized in Table 1. Most patients were in their 20s at this review. The median age at cancer diagnosis was 10 years (range, 0–18 years), and many of the patients have survived for a long time since their cancer treatment. In more than 80% of patients, anthracycline was included in the chemotherapy. The median cumulative anthracycline dose was 180 mg/m^2^ (range, 0–392 mg/m^2^), and exceeded 250 mg/m^2^ in five patients (15%). Half of the patients had a history of allogeneic HSCT. All patients who received irradiation to a field involving the chest were in the setting of total body irradiation for the conditioning regimen prior to HSCT.

### Cardiovascular findings

No patient had symptomatic cardiac complications at the time of the review. None of the patients required regular check-ups by cardiologists or medications for cardiovascular complications. Twenty-eight (82%) patients had routine blood examinations including measurement of the BNP level, and 29 (85%) had routine echocardiographic follow-up at the discretion of the physician. The laboratory and echocardiographic parameters are listed in Table 2.

**Table 1:** The demographic and clinical characteristics of the patients

	*N* = 34
**Age - Median (range)**,** years**	25 (20–40)
**Sex - No. (%)**	
Female	18 (53)
Male	16 (47)
**Cancer type - No. (%)**	
Acute lymphoblastic leukemia	15 (44)
Acute myeloid leukemia	9 (26)
Malignant lymphoma	5 (15)
Chronic myeloid leukemia	2 (6)
Aplastic anemia	1 (3)
Others	2 (6)
**Age at cancer diagnosis - No. (%)**,** years**	
≤ 5	8 (24)
6–10	10 (29)
11–15	12 (35)
≥ 16	4 (12)
**Age at referral to adulthood care - No. (%)**,** years**	
18–20	10 (29)
21–25	17 (50)
≥ 26	7 (21)
**Elapsed years from cancer diagnosis - Median (range)**	16.5 (5–30)
**Cancer treatment history**	
**Chemotherapy- No. (%)**	34 (100)
**Anthracycline use- No. (%)**	28 (82)
**Cumulative anthracycline dose - No. (%)**,** mg/m**^**2**^	
≤ 100	9 (26)
101–249	14 (41)
250–399	5 (15)
≥ 400	0 (0)
Dosage unknown	6 (18)
**Transplantation - No. (%)**	
Autologous stem cell transplantation	0 (0)
Allogeneic stem cell transplantation	17 (50)
**Irradiation including chest - No. (%)**	10 (29)

**Table 2:** Cancer therapy-related cardiac dysfunction parameters

	*N* = 34
**Symptomatic cardiac complications - No. (%)**	0 (0)
**LVEF** - median (range), %	61.3 (51.2–70.5)
**<50** - No. (%)	0 (0)
**E/e’** - median (range)	6.85 (4.3–12.3)
**> 8** - No. (%)	11 (32.3)
**GLS** - median (range), %	18.8 (12.8–25.1)
**<18** - No. (%)	11 (32.3)
**BNP** - median (range), pg/mL	6.84 (2–52.5)
**>18.4** - No. (%)	4 (11.8)

LVEF: left ventricular ejection fraction, GLS: global longitudinal strain

**Fig. 1:**
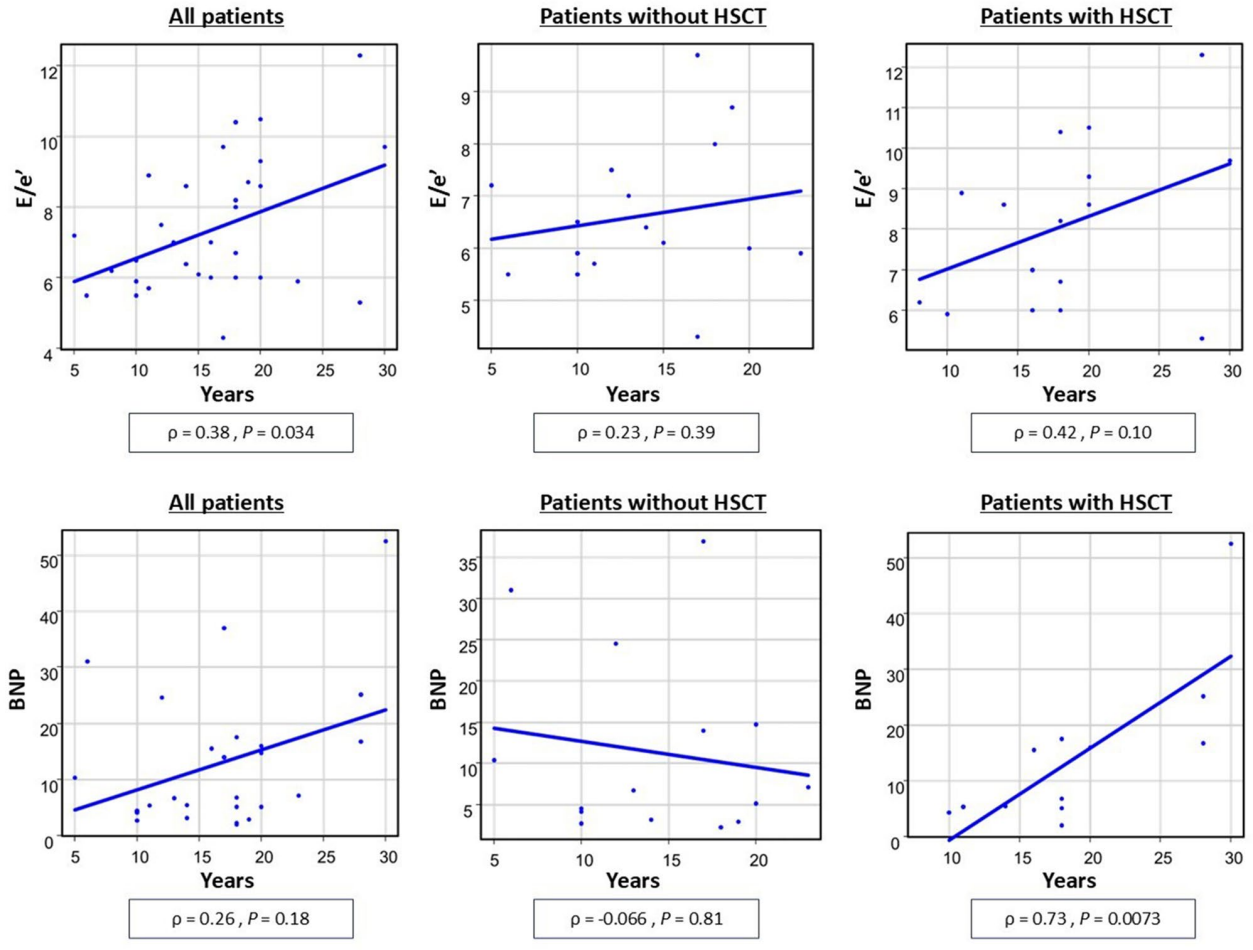
The correlation of the E/e’ ratio and the BNP level with duration since cancer diagnosis for all patients, patients who received HSCT, and patients who did not receive HSCT

The details of each parameter for each characteristic are listed in Table 3. EF, the E/e’ ratio, and the BNP level were higher in females compared to those in males; GLS was not significantly different. There were no significant differences for each parameter between the two groups based on age at cancer diagnosis (≤ 4 years or > 4 years). There were no significant correlations between age and each parameter. However, years elapsed since cancer diagnosis was positively correlated with the E/e’ ratio, although the correlation was weak (ρ = 0.38, *P* = 0.034) (Fig. 1). EF, GLS, and the BNP level were not significantly correlated with years elapsed since cancer diagnosis.

**Table 3:** Details of each parameter for each characteristic and treatment history

Variables		LVEF (%)		GLS (%)		E/e’		BNP (pg/mL)	
		Median (range)	P-value	Median (range)	P-value	Median (range)	P-value	Median (range)	P-value
**Sex**	**Female**	62.9 (54.1–70.5)	0.033	19.4 (12.8–23.2)	0.35	8.2 (5.3–12.3)	0.041	15.8 (4.5–52.5)	0.0015
	**Male**	59.4 (51.2–68.0)		18.4 (12.8–25.1)		6.2 (4.3–8.9)		4.4 (2.0–31.0)	
**Age at cancer diagnosis**	**≤ 4 years**	60.8 (58.5–63.4)	0.79	17.6 (12.8–22.5)	0.24	7.3 (5.3–12.3)	0.77	16 (7.1–25.2)	0.086
	**> 4 years**	61.3 (51.2–70.5)		19.2 (12.8–25.1)		6.9 (4.3–10.4)		5.4 (2.0–52.5)	
**Anthracycline cumulative dose**	**< 100 mg/m** ^**2**^	62.7 (57.3–68.0)	0.42	20.5 (15.6–25.1)	0.48	6.3 (5.3–12.3)	0.85	16.3 (4.1–52.5)	0.22
	**100–249 mg/m** ^**2**^	60.1 (51.2–68.0)		19.3 (12.8–23.2)		6.9 (5.7–9.7)		5.1 (2.0–37.0)	
	**≥ 250 mg/m** ^**2**^	61.1 (56.1–70.5)		18.7 (17.3–22.2)		7 (5.5–10.4)		11.2 (3.2–31.1)	
**Irradiation involving chest**	**No**	60.5 (51.2–68.0)	0.62	18.6 (12.8–25.1)	1	6.7 (4.3–12.3)	0.38	8.8 (2.2–37.0)	0.85
	**Yes**	60.5 (54.1–70.5)		18.4 (13.1–23.2)		8.2 (5.9–10.4)		5.4 (2.0–52.5)	
**HSCT**	**No**	62.2 (54.9–68.0)	0.42	18.9 (13.9–25.1)	1	6.25 (4.3–9.7)	0.040	6.7 (2.2–37.0)	0.35
	**Yes**	60.8 (51.2–70.5)		18.8 (12.8–23.2)		8.4 (5.3–12.3)		11.2 (2.0–52.5)	

LVEF: left ventricular ejection fraction, GLS: global longitudinal strain, HSCT: hematopoietic stem cell transplantation

In comparison by treatment history, the E/e’ ratio was significantly higher in patients who had HSCT than in those who did not undergo HSCT (median, 8.4 vs. 6.25, *P* = 0.040). No significant differences were observed in the EF (median, 60.8% vs. 62.2%; *P* = 0.42) or GLS (median, 18.8% vs. 18.9%; *P* = 1.00) between patients with and without HSCT. There was also no significant difference in the BNP level between patients with and without HSCT, but numerically higher in the HSCT group (11.2 pg/mL vs. 6.72 pg/mL; *P* = 0.347). In addition, there was a strong correlation between the years elapsed since cancer diagnosis and the BNP level in patients with a history of HSCT (ρ = 0.73; *P* = 0.0073), although there was no obvious correlation in the patients overall (ρ = 0.26; *P* = 0.18) or in patients without HSCT (ρ = -0.066; *P* = 0.81) (Fig. 1). The correlation between elapsed years and the E/e’ ratio, which was observed in the patient overall as described above, was analyzed separately for patients with and without HSCT, and a stronger correlation was found in the patients with HSCT (ρ = 0.42; *P* = 0.10) compared to those without HSCT (ρ = 0.23; *P* = 0.39).

Comparison by cumulative anthracycline dose showed no significant differences in EF, the E/e’ ratio, GLS, or the BNP level among the three groups: <100 mg/m^2^, 100–249 mg/m^2^, and ≥ 250 mg/m^2^ with doxorubicin equivalents. A history of chest irradiation did not affect these parameters.

## Discussion

In this study, we found that the E/e’ ratio was positively correlated with years elapsed since cancer diagnosis and was also significantly higher in CCS who had HSCT than in those who did not have HSCT. In addition, there was a strong correlation between years elapsed since cancer diagnosis and the BNP level in patients with a history of HSCT.

The E/e’ is the ratio of early diastolic transmitral flow velocity (E) to early diastolic mitral annular velocity (e′) [13], which has been widely used to estimate the left ventricular filling pressure and one of the indices of left ventricular diastolic function. The E/e’ ratio is known to be affected by aging [14]. However, given the ages of the patients in this study varied little, most were in their 20s, and no correlation with current age was observed, it is thought that elevation in the E/e’ ratio is affected by years elapsed since cancer diagnosis rather than by age-related changes alone. Some studies on the long-term follow-up of CCS have suggested that cardiac function deteriorates over time [15, 16]. A longitudinal study of pediatric acute lymphoblastic leukemia survivors reported that left ventricular contractile dysfunction was observed relatively early after chemotherapy and improved thereafter [16]. However, after a prolonged period (e.g., more than 10 years), restrictive physiology occurred. The increase in the E/e’ ratio seen in the long-term follow-up cases in this study may be reflective of restrictive physiology.

There have been several reports on the association between treatment history and cardiac diastolic dysfunction in cancer survivors. For example, anthracycline-induced late cardiac impairment was reported to manifest first as diastolic dysfunction before systolic dysfunction, and RT may also cause diastolic dysfunction as a late toxicity [15]. There are limited data regarding cardiac function in the long-term follow-up of CCS after HSCT, but HSCT has been reported to be associated with late cardiotoxicity in adults [5]. HSCT-induced cardiotoxicity is thought to be caused by a combination of various factors, including high-dose chemotherapy, irradiation, and immunosuppressive drugs [6]. Although there was no significant difference in each parameter in this study in terms of the cumulative dose of anthracycline, with or without RT, the significant increase in the E/e’ ratio in CCS treated with HSCT may still be because HSCT is one of the most intensive treatments that combines various toxic factors. The increase in the BNP level in patients who undergo HSCT and had survived for a long time may also reflect a subclinical cardiac burden due to late toxicity of this potent treatment. The subclinical changes observed in this study may serve as early indicators of symptomatic cardiac dysfunction.

In adult oncology, GLS is known to be a useful indicator for the early detection of CTRCD. Even in adult allogeneic HSCT patients, it has been reported that GLS is superior to EF for monitoring cardiac function [10]. However, most reports are based on evaluations during treatment or up to several years after the end of treatment [9], and its utility in long-term follow-up or in pediatric cancer survivors is unclear. In the setting of long-term follow-up of pediatric cancer survivors, diastolic indices including the E/e’ ratio or the BNP level which reflects the actual cardiac burden may be more useful.

There were no cases who had symptomatic cardiac complications in our study. However, some patients had echocardiographic or laboratory abnormalities, such as decreased GLS or an elevated BNP level; namely, they met the criteria for mild CTRCD in the ESC guidelines on cardio-oncology [11]. In CCS, severe, life-threatening health conditions as late toxicities associated with cancer treatment continue to increase with age [17]. Furthermore, cumulative incidence of cardiac events shows a marked increase particularly for survivors beyond the age of 35 years [18]. The cohort in this study was relatively young, with a median age of 25 years, which may be one reason why there were no cases who developed symptomatic cardiac events. In the future, some patients with asymptomatic CTRCD with abnormal laboratory or echocardiographic findings may develop symptomatic cardiac events as they age; thus, careful follow-up should be continued.

Comparison by patient characteristics showed that females had higher E/e’ ratio and BNP level than males, but EF was superior in females. It is known that there are sex-related differences in cardiac morphology and function [19]. Considering that the differences observed in the present study were inconsistent with respect to cardiac function, it may reflect conventional sex differences rather than differences in the susceptibility to cardiotoxicity risk.

Given that younger age at diagnosis (particularly age ≤ 4 years) is reported to be a risk for late cardiotoxicity [20], we divided survivors into two groups based on age at cancer diagnosis: ≤4 years and > 4 years, and compared each parameter between the groups. Although there were no significant differences in each parameter by age at cancer diagnosis, BNP tended to be higher in the patients who had treatment at younger ages. There were no differences in each parameter according to the cumulative anthracycline dose, with or without chest irradiation, which may have been due to the small sample size and young ages of the cohort in this analysis.

In assessing the cardiac status of long-term CCS who have transitioned to adult care, attention should be paid not only to the parameters of systolic function but also to those of diastolic function. The BNP level may also be useful in long-term survivors with a history of HSCT. In our hospital, we routinely focus on these parameters for long-term follow-up strategies in HSCT survivors. Furthermore, it is the most important to obtain detailed information about cancer treatment to ascertain the risk of late toxicity and to provide education to promote a healthy lifestyle to reduce cardiovascular risk factors as much as possible [21, 22].

This study had some limitations. First, this was cross-sectional study and included only small sample size; therefore, to more fully assess the effect of cancer treatment on late cardiotoxicity, longitudinal follow-up within the same cohort and a larger sample size is required. Second, we made comparisons between treatment histories only for CCS. To assess the effect of cancer treatment more accurately, it is desirable to compare the CCS with healthy controls of the same age group who did not receive any cancer treatment. Finally, this study was retrospective and most patients had a cancer diagnosis many years ago. Therefore, several cases lacked sufficient data on treatment history, such as cumulative anthracycline and radiation doses.

## Conclusion

Among childhood hematologic cancer survivors who have survived for a long time since their cancer diagnosis, particularly those who had HSCT, there was a significant increase in the E/e’ ratio and the BNP level. We need to pay attention to diastolic function and the BNP level in the long-term follow-up of CCS, particularly in those who had high-intensity therapies such as HSCT. To determine whether such subclinical abnormalities lead to symptomatic cardiotoxicity and serve as useful markers for the early detection of cardiac complications, further continuous follow-up is required.

## Data Availability

The datasets analyzed during the current study are available from the corresponding author on reasonable request.
